# Beyond the sentence: Shared reading within a high secure hospital

**DOI:** 10.3389/fpsyg.2022.1015498

**Published:** 2022-11-14

**Authors:** Megan Watkins, Kathryn Naylor, Rhiannon Corcoran

**Affiliations:** ^1^Institute of Health and Social Care, London South Bank University, London, United Kingdom; ^2^Faculty of Health and Life Sciences, University of Liverpool, Liverpool, United Kingdom; ^3^Mersey Care NHS Foundation Trust, Liverpool, United Kingdom

**Keywords:** therapeutic reading, high secure setting, psychological discourse analysis, longitudinal, case series

## Abstract

Ashworth Hospital provides care for inpatients detained under the Mental Health Acts who present a danger to themselves or others. Rehabilitative interventions can help support the best outcomes for patients, their families, care providers, and society. The efficacy of weekly Shared Reading sessions for four patients with experience of psychosis and a history of self-harm was investigated using a 12-month longitudinal case series design. Session data were subjected to psychological discourse analysis to identify discursive strategies employed to accomplish social action and change over the duration of the intervention. Archetypes of interactional achievement across sessions emerged. Broadening of capacity to consider was demonstrated through increased hedging and less declarative language. Increased assertiveness was achieved through reduced generalisation marked by a transition from second-person plural pronouns to more first-person singular pronouns. Avoidance of expression and disagreement strategies diminished over time. In addition, heightened engagement was accomplished through the increased tendency to employ functionally related and preferred responses within adjacency pairs, which mirrored non-verbal communicative strategies. Shared Reading shows promise for promoting the interactional accomplishment for individuals within high secure settings, who are ready to undertake a recovery-related activity. Pathways of interaction should continue to be explored, with consideration to the current study’s strengths and limitations. This study contributes to the understanding of efficacious reading study design and the interactional outcomes of therapeutic reading.

## Introduction

The Reader, established as a national charity since 2008, delivers Shared Reading groups across the United Kingdom and beyond, throughout diverse settings. The Reader’s Theory of Change suggests that the reading aloud of classic literature, guided by a facilitator, promotes the recognition and articulation of thought and feeling, thereby positively effecting outcomes including well-being, connectedness, and cognitive and affective flexibility. The Shared Reading model encourages participants to develop an understanding of the self and others, and to connect and realise change with breakthroughs signalled by the transition in language i.e., linguistic traces differing from the norm (Davis et al., 2016). Such transitions may be important given that undeveloped language skills hinder the mastery of self-control (Beaver et al., 2008) and perspective-taking (Rawn and Vohs, 2006). This is congruous with the notion that reading for pleasure can induce the state of “flow”; the experience of this state requires both control and concentration (Towey, 2000). The state of flow has been described as an “autotelic experience”, in which the actor’s attention is completely focussed on an activity and is not dependent on external goals or rewards (Csikszentmihalyi, 1975).

In terms of participant experience, Shared Reading aims to encourage balance, equity, and non-judgemental attitudes, and these aims may be a key component of Shared Reading’s efficacy, particularly within highly constrained environments and clinical settings. Individuals using health care services sometimes report not having adequate time to talk about how they are feeling and feeling pressured to agree with psychiatrists (Taylor et al., 2007). This may lead to failure to attend aftercare and negative expectations for therapy following discharge.

Well-managed Shared Reading sessions should provide an environment that allows patients/group members more time to consider and talk about how they are feeling than perhaps other creative interventions and provide a greater sense of continuity in the absence of an apparent “authority figure” than psychological therapies. In contrast to many existing psychosocial interventions, Shared Reading does not necessitate the direct and explicit repetition of an individual’s clinically relevant story which according to Hawton et al. (2011) can be perceived as unhelpful and distressing. Instead, Shared Reading tends to naturally elicit recollections of life beyond the rehearsed clinical narrative, uncovering and reviewing deeper, less emphasised episodes.

The discourse within Shared Reading group discussion prompted by literary material can be used to assess interactional skills and to indicate social psychological phenomena and self-development. Congruently, patients with a diagnosis of schizophrenia experiencing negative behavioural signs (e.g., flat affect, alogia, anhedonia, and avolition; Chang et al., 2018) have shown a tendency to flout social knowledge shaping conversational conduct (Corcoran and Frith, 1996). Concerning relevance, adjacency pairs have been considered the most basic conversational unit, a two-part exchange between two speakers that provides speakers with a frame of reference for conduct to achieve inter-subjectivity (Taguchi, 2019). Turn-taking can take many forms, for example, greeting/greeting, information/acknowledgment, and accusation/denial (Qodriani and Wijana, 2021). It has been suggested that disrupted turn adjacency does not always lead to incoherent interaction although there have been limited suggestions as to how exactly coherence is maintained in such cases (Berglund, 2009).

Co-construction of discourse can establish a sense of community. A discourse analysis of an online graduate course identified how patterns of agreement were linked to shared understanding and an enhanced sense of cohesion (Lapadat, 2007). Sophisticated social negotiations were found to allow disagreement whilst maintaining community which was achieved through allowing an opportunity to face-save, showing understanding, softening, and balancing. In addition, humour was used, participants invited comments, employed inclusive language, showed alignment with other participants, and used familiar genres. These findings indicate that growing social capital within groups allows communication of mutual benefit.

Furthermore, discourse can drive behavioural change. Three dominant devices achieving mobilisation and public engagement were identified using a psychological discourse analysis of two Facebook event pages (Sneijder et al., 2018). Positive atmosphere and “togetherness” were promoted through the use of positive language to undermine or attenuate negative event aspects while the use of the pronoun “we” constructed collectivity. In accordance, the use of “we” in discourse can be used to promote solidarity, shared authority (De Fina, 2003), and construct identity, for example, through indexing inclusivity and exclusivity (Selvi et al., 2021). Additionally, “we” can also introduce ambiguity by hiding agency and has been employed for this purpose in controversial speech (Jalilifar and Alavi, 2011). Flexible use of “we” can influence the power dynamic through, for example, the representation of professional and subgroup positions (Kvarnström and Cedersund, 2006).

Similarly, the use of the singular first-person pronoun “I” can have a multitude of different rhetorical effects. An investigation of a corpus of congressional speeches reported that the functions included achieving self-focus, the exhibition of dominance, to express strong opinions, in turn, dismissing others’ opinions, to show compassion, to express personal wishes, and to narrate a story (Lenard, 2016). In studies of power and political discourse, the use of “I” has been associated with declaring responsibility, strong conviction, and willingness to take risks.

The importance of discourse type and implicit alignment of language has been highlighted in the literature. In a discourse analysis of nine individuals’ first psychotherapy sessions, interlocutors most frequently adopted colloquial discourse, whereas the therapists mostly used therapeutic discourse (Wahlström, 2018). It was reported that the common expressions used and shared by therapists and service users within the session allowed for intimate experiences to be explored from new perspectives, and the frequent use of colloquial discourse demonstrated how the person-to-person relationship was a primary function of sessions. In contrast, over-lexicalisation may have a redundant effect, and chaos of stories and events may result in confusion. The discomfort it generates has been linked to countertransference within therapy sessions (Castells, 2018).

The use of silence, the antithesis of over-lexicalisation, has been investigated as a tool (Ephratt, 2008, 2012; Nikolić, 2016) within both conflict management and psychotherapy. Chowdhury et al. (2017) suggested that silence can indicate hesitation or indecisiveness of the speaker and may be used to force another speaker to respond. Qualitative analyses examining therapist perceptions of the use of silence in therapy found silence was used to show empathy (also recognised as a communicative resource; Martinovski, 2006), facilitate reflection and expression, and encourage clients to take responsibility (Ladany et al., 2004). Furthermore, therapists perceived their use of silence to be positively associated with the experience of providing therapy. Therapists, however, reported that they did not tend to employ silence as a communicative strategy with individuals experiencing psychosis, anxiety, or anger. The appropriateness of employing silence and other rhetoric devices appears, therefore, to be client and activity-specific.

The use of therapeutic arts–based therapies has received greater acceptance and recognition from health care professionals and the public in recent years, due to a growing body of evidence supporting outcomes such as enhanced social connection and awareness (National Collaborating Centre for Mental Health UK, 2014). This has led to investment in mental health research networks drawing together professionals from the sciences, humanities, and arts (Medical Research Council, 2018). Much of the research assessing Shared Reading within clinical populations has been conducted within community settings and with predominantly female samples. Shared Reading may be particularly beneficial within high-security psychiatric settings through its potential to improve the quality of interactions and thus level of connectedness.

The goal of this study was to uniquely investigate: (i) participants’ use of discourse to accomplish social action across Shared Reading sessions, specifically employing psychological discourse analysis; (ii) to do so within the context of a high secure setting, both drawing on the existing literature and allowing the identification of new, perhaps context-specific pathways of interaction; and (iii) to employ a case series design to identify and differentiate stylistic tendencies and person-centred change over time.

## Materials and methods

### Design

A 12-month case series design investigated the efficacy of weekly Shared Reading for patients at Ashworth Hospital. Ashworth Hospital is a National Health Service hospital in North West England for patients requiring care and treatment in high secure conditions. The study was reviewed and approved by North West—Liverpool East Research Ethics Committee (Reference 17/NW/0114).

### Data collection

The sessions were in keeping with the Shared Reading model. Sessions took place over 2 h, with a short break mid-session. Usually, both a story and a poem were read aloud and discussed within the session. A record of the material can be found in Table 1. Reading material was selected by the facilitator, and researcher from literary fiction resources recommended and provided by The Reader. Sessions were facilitated by an Associate Specialist in Forensic Psychiatry [KN] who is a trained Shared Reading group leader and the researcher [MW] who also completed a Read to Lead course provided by The Reader and attended all sessions.

**TABLE 1:** Record of sessions and material forming corpus for analysis.

Date from 2017	Group number	Attendees	Material read
7th September	5	Clive Patrick John PN004 PN005	A Selection from “Three Songs at the End of Summer” by Jane Kenyon
12th October	4	Clive John PN005 Max	“Penny in the dust” by Ernest Buckler and “The Stone Beach” by Simon Armitage
9th November	4	Clive Patrick John Max	“Faith and Hope Go Shopping” by Joanne Harris and “Let me die a youngman’s death” by Roger McGough
7th December	5	Clive Patrick John Max PN005	“Christmas Cracker” by Jeanette Winterson and “Christmas Light” by May Sarton
18th January	4	Clive Patrick John Max	“The Loss” by David Constantine and “Entirely” by Louis MacNeice
15th February	4	Clive Patrick John Max	“Beyond the Bayou” by Kate Chopin and “The Journey” by Mary Oliver.
29th March	4	Clive Patrick John Max	“Good-for-Nothing” by Dic Tryfan and “Bluebird” by Charles Bukowski
26th April	4	Clive Patrick John Max	“Two Gentle People” by Graham Greene and “Along the Road” by Robert Browning Hamilton
17th May	2	John Max	“Miss Brill” by Katherine Mansfield and “Alone” by Maya Angelou
7th June	2	John Max	“The Bull” by Saki and “Trust” by D. H. Lawrence

Sessions took place in a therapy suite within Ashworth Hospital and the researcher audio and video recorded all sessions. Participants were invited to a taster session prior to study commencement to help participants decide whether they wished to participate and informed consent was sought prior to starting the study.

The data sources comprised 39 videos and audio-recorded Shared Reading group sessions (approximately 55 h of discourse). The data to be analysed were selected, generating the corpus; salient sessions were selected by the researcher, in agreement with the facilitator and wider research team. Sessions that were considered salient best addressed the research goals and were attended by regular participants allowing change to be observed over time. Transcription, utilising both audio and video recordings, was performed by the researcher to allow full immersion and to respect the sensitivity of the data. All files were accessed and stored using the researcher’s password-protected Mersey Care NHS Foundation Trust or University of Liverpool account, and participant responses were pseudo-anonymised.

### Analysis

A psychological discourse analysis, a discursive psychological approach proposed by Goodman (2017), was employed to analyse sessions. The analysis procedure was, in keeping with methodological recommendations, focussing on how discursive and rhetoric devices are implemented to accomplish social actions. As advocated by Willig (2001), the researcher also adopted a critical stance and demonstrated awareness of the social context. The epistemological foundations of discourse analysis are within social constructionism rather than positivism which is concerned with uncovering the true nature of actions (Johnstone, 2002). Appropriate research questions were generated that were in keeping with ensuing analysis and discursive theory. The focus was on how participants interacted with the reading material and group members and did not centralise around speakers’ thought processes or attitudes toward a topic of discussion.

A “simplified Jeffersonian” (Goodman, 2017) level of transcription was undertaken for reader accessibility. Transcription contained sufficient but not unnecessary detail to address the research questions. Body language and pauses were noted when affecting the meaning of discourse. Transcripts were line numbered for clarity and ease of referral. A preliminary rereading of transcripts was undertaken for data familiarity. Action orientation, i.e., what was being achieved by interaction and initial thoughts were recorded through marking and annotation of transcripts. Drawing on the vast literature, discursive and rhetoric devices used within discourse were identified, for example, use of language of certainty, endorsement seeking, turn-taking, and strategies for disagreement. The use of repertoires, ideological dilemmas, and how subject positions and identity were constructed by the speakers was examined. Devices were recorded through marginal writing on transcripts. Strategies that best addressed the research questions were selected, and extracts and examples were collated in a word document. The extracts were described to illustrate cases for each participant.

### Participants

Initially, ten male participants were recruited, for this year-long Shared Reading intervention. Over the course of that year, the attrition rate was 60% leaving four regular participants upon whom this case analysis is based. Two other participants attended 23 and 5% of the sessions before withdrawing. The case studies present discourse archetypes and participants represented a complex forensic sample; all participants had experienced psychosis, had a history of self-harm, and most had been in the prison system. Participants referenced troubled childhoods, problems at school, and were involved in crime from an early age. These particular men had less of a problem with substance use than the general clinical/forensic population but all of them had experienced it at some point. None had the experience of full employment, two participants regarded themselves as readers prior to the study, two did not, and two participants experienced neurocognitive impairments that impacted their ability to concentrate. The participants shared similar demographic characteristics such as age (*M* = 45.25, *SD* = 6.45) and ethnicity, all were White British. Each of the four participants attended over sixty percent of sessions, and reasons for occasional non-attendance of regular participants were mostly attributable to physical illness or other appointments. Discontinuation of two participants beyond the 25th session was due to external, non-study-related factors such as service transfer and/or logistical issues.

## Results

Pseudonyms are employed for the following cases.

### Participant one—Clive: A broadening of capacity to consider alternative interpretations of events

Clive attended 24 out of 39 study sessions and was present for eight out of ten sessions forming the corpus for analysis. The participant did not generally require encouragement to speak, took more turns than other speakers, and his contribution was generally descriptive. Clive’s discourse demonstrated a broadening of capacity to consider different interpretations across sessions and over time. This change was demonstrable both in response to the text and in response to the opinions of other group members to some degree. Particular discursive devices, the change in use and culmination was identified as illustrating this enhanced capacity. These were predominantly the use of certainty and declarative language, consensus, polysyndeton (the use of successive conjunctions), appeals to the listener, and posing of substantive questions.

Clive’s discourse in the first few sessions was characterised by expressions of high certainty and commitment to his initial interpretations. This was evident in Clive’s discourse around the characters’ thoughts, feelings, and actions. For example, “she’s got to…” and “erm that’s still er that’s still basically the same” (session one p. 29 line 13 and p. 44 line 28), “he must be thinking…” (session two p. 18 line 21), “that’s the way it should be” (session three p. 17 line 16), and “I think she’s doing what I said before… she feels reborn again” (session four p. 59 line 15).

Over time, there was a move to greater use of hedging phrases and words associated with less certainty; “it means to have I think it means to have like erm…” (session five p.12 line 38), “so I think looking at that only by my own experience…” and “could be loads of different things” (session five p. 18 line 22). Clive’s use of hedges served to show his improved consideration of different points of view and seemed to convey a degree of humility by reducing the force of his statements. At six months into the intervention, Clive showed some recognition of this; “I think so anyway probably just prove me wrong as we get further along that’s the way these stories are” and “I’ve changed my mind now about that…” (session six p. 6 line 2 and p. 42 line 13). Furthermore, Clive displayed some self-corrective language in session eight (p. 16 line 26); “so it’s the be- it might be the beginning of a little affair mighten it because you’ll alwa- probably say that may happen or you might….”

Toward the start of the intervention, Clive’s discourse was characterised by the use of first-person plural pronouns which appeared to act as an indicator of general agreement and in doing so reinforced the speaker’s own interpretation. An example of this use of “we,” its pairing with the intensifier “all” (a quantifier used for emphasis), “ourselves,” and the use of “we” within a rhetorical tag question was evident within the discussion of *A Selection from Three Songs at the End of Summer* by Jane Kenyon, session one (p. 4 line 15): “and I think I think we’ve all stood under a tree and to protect ourselves from rain and she can feel that rain dripping down off from the tree so her stepping out in the rain…” and “we’re talking about a pretty big nest here aren’t we.”

**FIGURE 1:**
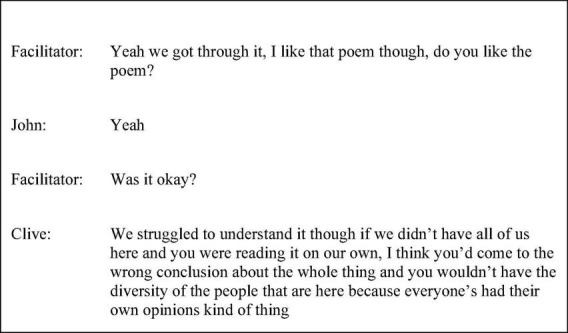
Session two extract (p. 45 line 17).

Clive’s use of polysyndeton, specifically the successive use of “and,” elongated the discourse. The use of the transition “so” further focussed the attention of the listener, before drawing a conclusion. Whilst “I think” can serve as a hedge, contextually, given its repetition, coexisting devices, and syntactic placement as a preface, the effect in this study may be rather factive accomplishing emphasis. Toward the end of session two, as shown in the transcript excerpt in Figure 1, the use of “we” and “all of us” was initially used to speak on behalf of the group when Clive conveyed his difficulty in interpreting the material. However, this was not sustained throughout the utterance given the adoption of the second-person plural “you” paired with the modal verb “would.” This granted genericity and attenuated agency. This was followed by an explicit acknowledgment that members of the group had different opinions. In contrast to the session one example, “I think” increased in hedging function, embedded within the utterance. The complement “because” whilst drawing a conclusion had a less exertive force when followed by the terminal tag and hedging phrase, “kind of thing” ascribing less certainty.

**FIGURE 2:**
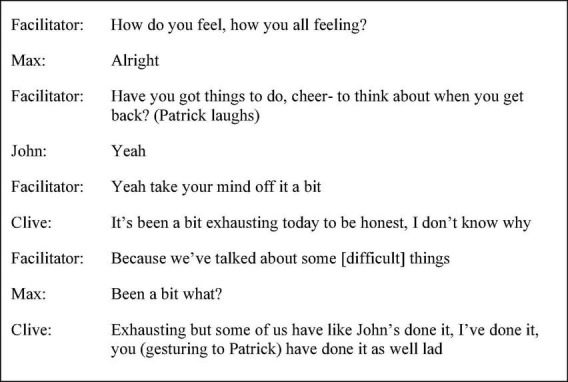
Session six extract (p. 44 line 5).

The facilitator, acknowledging sensitive discussion within session six, as shown in Figure 2, proceeded to “check in” with participants as part of a debrief before participants returned to the ward. Patrick laughed in response to the facilitator’s question, “Have you got things to do, cheer- to think about when you get back?” Whilst laughter perhaps served to indicate amusement at the false start and anticipated understatement (cheer you up), on another level, it functioned to terminate talk acting as a turn rejection. John and Max’s single-word neutral responses, “yeah” and “alright,” respectively, did not require expansion and functioned to push the interaction forward. In contrast, Clive conveyed, although with referential ambiguity, that reflection and disclosure within the session had been cognitively demanding.

Clive’s use of the adjective “exhausting” was accompanied by a hedge and honest statement to convey a personal rather than communal record in addition to the anticipatory self-identifying and face-saving expression, “I don’t know why.” Upon Max seeking clarification, Clive expanded not through the use of inclusive first-person plural pronouns but using address terms, the singular first-person pronoun “I” and the singular second-person pronoun “you,” forming a three-part list to augment the idea and separate agency. The approximation and hedge “some of us have like” paired with the ambiguous verb construction “done it” (i.e., spoken about difficult things), and the informal terms of address, “lad,” softened the discourse and served to portray a group of individuals with social actions in common, as opposed to signalling a single body all with the same experience. In this way, Clive established a form of collectivity as opposed to his prior tendency to prematurely proclaim an established consensus.

Over the duration of the intervention, Clive’s discourse demonstrated a shift in framing from the tendency to be speaker focussed to more listener-focussed. For example, in session one, the use of cajolers such as “can I just say something” (p. 34 line 26) and “you know” served as appeals to the listener and turn-entry devices allowed structuring of the conversation; “you know what, that’s where man- a lot of people don’t know this- that’s where man actually learnt to sing” (p. 5 line 29). The modal “actually” conveyed information about the attitude of the speaker with regards to the message, communicating the speaker’s view of the utterance’s unexpected content, novelty, and certainty about the surprising content. This was reinforced by the aside “a lot of people don’t know this” which has an interactive function, relating the topic to an everyday frame and marking the digression, whilst Clive also established himself as a source of superior knowledge in the group. In session three, Clive continued to convey his own interpretation of the text with appeals to the listener such as “isn’t he…you can tell…,” however, the use of an option marker “or” also showed consideration for another speaker’s turn; “he’s wishing he’s wishing isn’t he that you can tell by because he mentions death so much I think he’s scared of actually dying not just a youngman’s death but he’s scared of dying in general or like you said he wants to be able to have that opportunity to be able to do the things that he might never of done just faded into the night kind of thing…” (p. 7 line 14).

**FIGURE 3:**
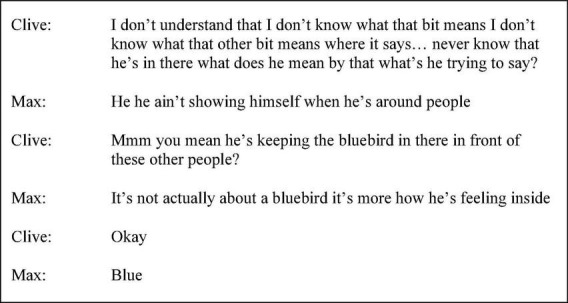
Session seven extract (p. 67 line 24).

Clive’s discourse in session four demonstrated further alignment and recognition of another speaker’s turn, the use of “well” demonstrated receipt of information whilst “I mean” promoted speaker clarification; “well yeah you’re right you’re bang on the button there [Facilitator]…‘cause I can remember… I couldn’t cope I mean absolute- I was my most depressed…” (p. 63 line 9). The frequent use of the singular first person “I” contributed to the reflective stance and heightened self-involvement through conveying individualised, personal experience. The tendency for listener-focussed speech in later sessions was evidenced by Clive’s use of substantive questions. In session one, Clive’s discourse was, at times, directive and knowledge testing creating a demand for certain responses and exercising social control, for example, “there you are [name], there’s a question for you - what’s a gathering of crows?” (p. 7 line 2). Later discourse was more enquiring, “if someone said I’ll give you a hundred quid to do it again would you do it?” (session six p. 14 line 8). Clive’s discourse in the extract from session seven, shown in Figure 3, during discussion of *Bluebird* by Charles Bukowski exerts no constraints on the following turn and is knowledge-seeking rather than knowledge-giving.

Over the sessions, Clive demonstrated a shift from expressions of high certainty to less certain language and developed a more explorative style of questioning. This indicates how flexibility of thought can arise through participation in Shared Reading and how this promoted development of connectedness with other group members, in keeping with the Theory of Change.

### Participant two—Patrick: Increased assertiveness

Patrick attended 25 out of 39 study sessions and was present for seven out of ten sessions forming the corpus for analysis. Patrick’s discourse displayed increased self-confidence and assertiveness across sessions, which could be evidenced through changes in generalisation, voicing disagreement in the interpretation of the text, endorsement seeking, and use of humour.

**FIGURE 4:**
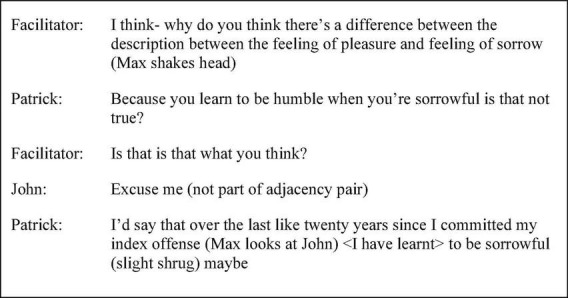
Session eight extract (p. 40 line 3).

Patrick’s discourse up until five months into the intervention showed a tendency to employ plural second-person pronouns, conveying personal experience and opinion through generalisations about how people feel without a clearly identifiable referent. In session one, the facilitator posed the question, “what was it like waiting to go on that first day [of school]?” (p. 32 line 7), to which Patrick responded, “it’s like you’ve got no choice” (p. 32 line 9). Similarly, ambiguity of agency was achieved through discourse such as “when you’re a kid all your life’s like on rails isn’t it” (session one p. 41 line 31) and “for a lot of people in here it’s a bit depressing” (session four p. 55 line 24). At five months into the intervention, a story called *The Loss* by David Constantine was read, in which the character Mr. Silverman loses his soul. Patrick’s use of self-reference uniquely and unambiguously conveyed access to the speaker, using more first-person singular pronouns. For example, “I think I was there at one point many years ago I was like that at one point… no joy … feelings nothing” (session five p. 30 line 39). In session eight, as shown by the extract in Figure 4, the agency behind the generic “you learn” was revealed when Patrick drew on personal experience when prompted, expanding his turn with the use of “I.” “I” as the subject of verbs portrayed a truthful narrator and increased level of ownership over discourse albeit then attenuated with the use of the hedge “maybe”.

Patrick’s discourse was initially characterised by questions and hesitant tonality, “[Researcher]… is it in America?” (session one p. 21 line 8), “are they old people?” (session three p. 17 line 3), “is he actually thinking them thoughts now the dog?” (session four p. 6 line 30), and “do you think she’s found someone to love?” (session four p. 61 line 20). Posing utterances as questions accomplished conveying personal interpretation in an unassertive, unchallenging manner. Uncertain language and use of tag questions contributed to this effect; for example, “he wants to die young but he doesn’t if you understand what I mean” (session three p. 7 line 8). The contrastive marker “but” adds lexical ambiguity and contradiction whilst the tag question “if you understand what I mean” relied on meta-knowledge of the listener and served to seek endorsement. In later sessions, Patrick communicated interpretation through more declarative utterances.

**FIGURE 5:**
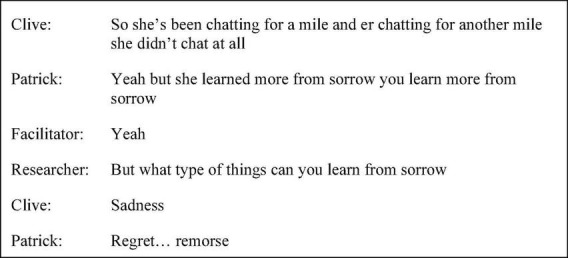
Session eight extract (p. 41 line 13).

Disagreement with other speakers was managed diplomatically with the use of hedging; for example, “I don’t think it’s… a bird as such” (session seven p. 69 line 5), “I think he it’s not necessarily what country I think it depends on the person as a person” (session seven p. 38 line 17), and “feels worthless as well because she’s got nothing to do…” (session eight p. 30 line 9). The extract from session eight, as shown in Figure 5, provided a further example of how Patrick more assertively expressed opinion and feeling; “yeah” served to acknowledge the previous turn whilst the contrastive marker acted as a rejecter and successive repetition of Patrick’s utterance reinforced the speaker’s message. Patrick proceeded to demonstrate the development of the emotional lexicon, describing how you can learn “regret” (and then deeper), “remorse” from sorrow.

Additionally, Patrick’s use of humour and portrayal of characters in initial sessions generally drew parallels with the experience of psychosis, serving a somewhat self-depreciative function; for example, “I think they’d have something to say if we go off on adventures here” (session three p. 39 line 26) and “he’ll end up in here won’t he” (session four p. 23 line 23). In session eight, upon the researcher drawing attention to word selection within the poem *Along the Road* by Robert Browning Hamilton, “it’s interesting how the word chattered was picked… why chattered” (p. 45 line 30), Patrick responded through an impersonation of the imagined character, “a word for rambling (p. 46 line 6)…oh this is great this is good this is brilliant (laughs).” In contrast to previous humour, the discourse was not negatively inflected and demonstrated the embodiment of the character rather than a comparison to personal circumstances. The shift in positioning suggests heightened absorption in the material and was accompanied by a notably animated tone, which emerged concomitantly with decreased hesitancy.

In summary, over the sessions, Patrick developed discursive strategies to increase the level of ownership of his discourse; his emotions and thoughts became public. Patrick showed greater confidence in expressing his own opinion and interpretation of the reading material. Indeed, the Theory of Change describes how both the reading material and facilitator can enhance the articulation of thought and feeling. This in turn led to greater assertiveness, a key social skill and diminished fear of threat to the self from exposing feelings and the self.

### Participant three—John: Decreased avoidance

John attended 32 out of 39 study sessions and was present for all ten sessions forming the corpus for analysis. John’s discourse was characterised by particular devices: alignment, repetition, disclaimers, and avoidance. The extent to which communicative strategies served self-presentation and monitoring functions attenuated moderately over time. The quantity and turn-taking frequency of John’s discourse varied considerably between sessions but generally increased.

**FIGURE 6:**
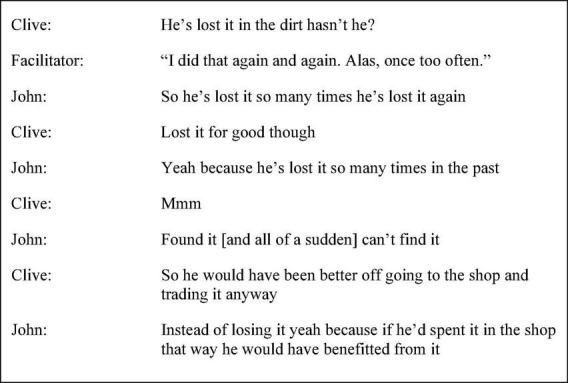
Session two extract (p. 12 line 16).

John’s discourse in the first six months was particularly marked by repetition and paraphrasing of other speaker’s turns, with a tendency to follow and align, particularly with Clive; “like Clive says… Clive what were you going to say” (session one p. 30 line 26) and “agree with you Clive good stuff” (session five p. 27 line 46). Similarly, in session two, as shown in Figure 6, John repeated the idea that a character in the story *Penny in the Dust* by Ernest Buckler was embarrassed upon losing a special penny from his father. John’s use of “so” and “because” continued to reiterate and reinforce an established idea with the use of “yeah” also serving to align with Clive. Subordinate responses within adjacency pairs, through the repetition of established ideas, functioned to avoid expansion and disagreement. The use of non-committal language also served to avoid expressing a personal opinion, for example, in session three following the facilitator’s question, “do you think they go together the poem and the story?” (p. 37 line 3), John responded, “might do.” This tendency was to some extent acknowledged by John in session ten (p. 24 line 6); “when I don’t make comments it’s because I don’t understand it properly…today I’ve understood quite well…when I know what I’m doing when I’m working it out that’s when I comment a lot…because I understand it and I understand what it says and what it’s about.”

John’s discourse also reflected positive self-portrayal; “I remember I remember everything from the age of two” (session one p. 3 line 16), “I went to the dentist this morning and [they] said I had a good set of strong teeth” (session three p. 19 line 5), and “and like I say I’ve been here six years I’ve done some therapies and I must have benefitted off them because I’ve not self-harmed” (session four p. 47 line 18). When discussing the number of sessions completed in session five, John enquired as to whether attendance was recorded on the medical record system, “is it on PACIS is it on PACIS?” (p. 52 line 45). Monitoring self-presentation was also accomplished within discussion through John’s use of disclaimers, such as “I don’t hear voices no more you know but… it’s been right as rain” (session six p. 32 line 22) and “like I said everybody’s got a good side and a bad side haven’t they like I’ve never lashed out at anybody since I’ve been here you know ‘cause I’m not a bad person you know what I mean…” (session seven p. 52 line 5).

John went on to explicitly acknowledge concern with self-image, “I was scared I was worried about what people thought of me personally I use to to er worry about what people would think of me…I do think because I’ve been in the nut house…” (session seven p. 56 line 16). John’s attitude was strongly conveyed by the use of derogatory epithets.

**FIGURE 7:**
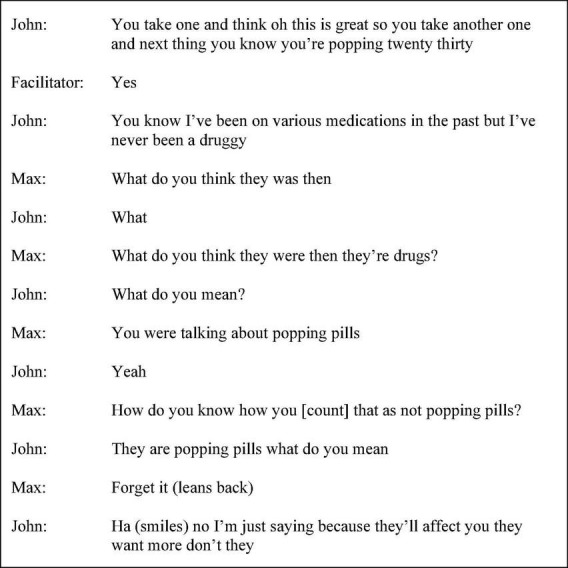
Session seven extract (p. 36 line 16).

John employed topic change to accomplish avoidance which appeared to be a sophisticated strategy for managing the direction of the conversation, albeit potentially maladaptive in the context of therapeutic encounters. For example, whilst John disclosed the death of a relative following the misinterpretation of the previous speaker’s prompt, John proceeded to reject empathy and prevent expansion through talk termination, “time for a drink I think time for a break” (session six p. 23 line 25). Non-alignment in footing (i.e., speaker selection and changing of context) during an interaction with Max was also used to avoid voicing a demanded response, shown in Figure 7.

Footing placed the speaker in the least self-threatening position, accomplishing to nullify and disengage from Max’s notion that taking prescription drugs for non-prescriptive purposes was not too dissimilar from the “druggy” behaviour John disaffiliated from, “I’ve never been a druggy” (session seven p. 36 line 20). An interlocutor seeking clarification for a question they do not understand and providing an irrelevant response seems to be an evasive strategy. However, John continues to question the question posed and responded by changing the textual content, following the receipt “ha” and rejection, “no.” The interpretation in which the speaker avoids discourse that is dis-concordant with positive self-representation is, in this instance, more in keeping contextually with the surrounding discourse than interpreting the exchange as merely a misunderstanding. Similarly, in session ten, when the facilitator remarked upon the discussion of familial trust, “but does that mean that people would automatically trust?” (p. 47 line 24), John responded, “I would like them to trust me yeah ‘cause I’m their father” (p. 47 line 25). John did not align with the facilitator’s positioning as indicated by the contrastive marker “but” and used the modal verb “would” and “yeah” to acknowledge the previous turn and topic shift, achieving a degree of evasion. Whilst the maxim of relation, one of Grice’s (1975) four maxims forming the cooperative principle remained somewhat violated in this example, the speaker did not employ complete avoidance strategies or ambiguous language.

The novelty of the speaker’s turn contrasts the imitation and alignment devices that exemplified earlier sessions. In summary, over the sessions, John’s interaction style became less characterised by the tendency to follow within an adjacency pair and the extent to which discursive devices monitored self-presentation reduced to some degree. Change in avoidance strategies may indicate a greater openness to other experiences, which the Theory of Change describes as “breaking through.”

### Participant four—Max: Heightened engagement

Max attended 30 out of 39 study sessions and was present for nine out of ten sessions forming the corpus for analysis. Changes within adjacency pairs, strategies for disagreement, non-verbal behaviour, and disclosure served the purpose of heightening social interaction and engagement over time. Generally, Max’s discourse reflected literary knowledge. Max was often able to add to the group’s understanding of settings within the material read and biographical information about authors.

In session two, the facilitator provided introductions upon Max starting the intervention. Following greetings from both the researcher and Clive, the facilitator enquired, “[Researcher] comes from the university like I explained and do you know Clive?” (p. 1 line 23) to which Max responded, “Who’s Clive?” (p. 1 line 25). Max’s utterance did not attend to cues provided by previous adjacency pairs. In addition, verbal acknowledgment of the other speaker’s actions or presence was absent resulting in abruptness of turn. Similarly, when the researcher asked, “do you think his dad might be upset that his son thought [that]” (session two p. 22 line 1), Max provided a non-sequitur, boundary-challenging response, “Are you from London?” (p. 22 line 4) which may also reflect distraction from the session. The dis-preferred nature of discourse was emphasised by interactional differences in framing.

**FIGURE 8:**
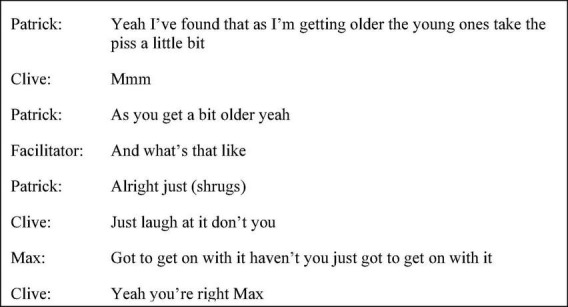
Session six extract (p. 29 line 12).

A later instance in this session demonstrated Max employing functionally related adjacency pairs but in doing so Max dismissed other group members’ interpretation of the material, “you aren’t going to have six girlfriends are you?” (p. 31 line 27). In contrast, Max used a different discourse style for managing disagreement within a discussion about the effects of money toward the end of the intervention. When John suggested, “too much money goes to people’s heads,” Max responded, “don’t think she’s one of them though she’s erm she’s quite (looking to facilitator) is it corpus mentis [*sic*]?” (session nine p. 35 line 27). The hedge phrases “don’t think, quite,” the contrastive “though” and hesitator “erm,” served to tentatively soften the rejection. In addition, the aiding of group inter-subjectivity in the latter sessions was more collaborative in style; Max was more interactionally responsive to group members; “yeah he is he’s a poet and an author” (session seven p. 52 line 20) and conveyed access to other speakers’ mental states through empathic turns. For example, in session six, as shown in Figure 8, Max acknowledged previous turns discussing ward dynamics and aligned with Patrick and Clive’s non-verbal and verbal behaviour. Continuation of sentiment and experience could be identified through the endorsement-seeking tag question, “haven’t you,” recurrent employment of “just” mirroring previous turns and Max’s successive repetition of “got to get on with it” which produced an amplifying effect.

At this six-month point, the use of adjacency pairs served to promote collegiality. Max’s language showed a change in attitude, particularly toward poetry, across the intervention. In session two, negative sentiment was expressed through negation, “[got to be honest with you] I’m not really one for…poems don’t really [get] reading them” (session two p. 30 line 14). This contrasted the engagement within session eight, “so it’s totally opposite to the first paragraph isn’t it” (p. 39 line 27) and “I tell you… it’s a way of explaining how he feels” (session seven p. 69 line 6). The use of “so” serves to indicate Max drawing a conclusion with the use of the intensifier “totally” resulting in a more animated style of discourse. The use of cajolers such as “I tell you” also served to indicate more listener-focussed interaction. Furthermore, positivity was expressed explicitly, “I actually like that one” (session seven p. 71 line 8) and “it was a pleasure today I enjoyed it” (session eight p. 49 line 5).

**FIGURE 9:**
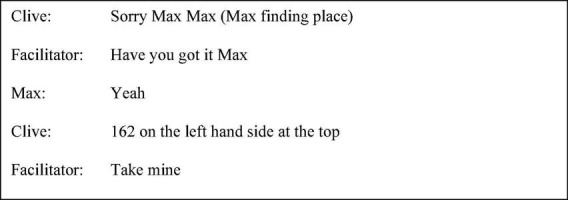
Session seven extract (p. 10 line 2).

Throughout the intervention, Max demonstrated an increasing effort to reengage with material and interaction when concentration or engagement lapsed. For example, session four was marked by body language indicating disengagement and distraction such as nail biting (p. 34 line 4), moving the chair back (p. 70 line 11), and fidgeting (p. 63 line 17). Whilst Max remarked, “I’m tired” in session five (p. 3 line 4) this was followed by Max sitting up, making a concerted effort to re-focus. Accordingly, this was mirrored within verbal communication, “can we get a drink in a minute can we get a drink in a minute… what’s that… what’s that (session five p. 25 line 17).” Max’s frustration at losing his place during reading of the material was recognised by other group members and evident in an extract from session seven, shown in Figure 9.

Change in disclosure and expression of feeling was observed in Max’s discourse. Following disclosure of the loss of relatives at a young age in session three, the discourse was marked by a long weighted pause marking both listener empathy and speechlessness. Disclosure appeared more like a revelation prompted by the poem as opposed to routine or confessional. John’s starter “well” (p. 11 line 28) remained an incomplete phrase whilst Clive communicated empathy more explicitly, “Too early that isn’t it too early they sa-” (p. 11 line 29). Talk was terminated by Max’s response, “well having said that it was so long ago I was so young I didn’t really know what was going on” (p. 11 line 30). The use of “well” functions to preface a topic shift, marks an insufficient response (i.e., not the response intended by the previous turn), it rejects empathy given and in doing so avoids expansion.

Like the literature itself, the participants’ discourse was full of spaces for inference, potential resonance, and other unspoken words. However, Max did expand description of negative experiences in later sessions; “I’ve been like that as well… when I was in The Scrubs I wouldn’t say I was dirty… but er I didn’t wash myself I didn’t care about myself I didn’t eat” (session five p. 31 line 1). Upon reading, *Two Gentle People* by Graham Greene, the group discussed the nature of communication that you may have with a stranger. Max’s discourse explicitly communicated discomfort with conveying feelings, heightened through hesitation and endorsement-seeking appeals to the listener, “I don’t like that me I’m … I’m quite on my own if you know what I mean I don’t really express my feelings you know what I mean (session eight p. 12 line 26).”

Similarly, in session nine, participants discussed what they would do with a million pounds, to which Max responded, “do you know what I’d do… I want to build my own prison … because I’d feel safe” (p. 29 line 24). In light of this discourse, prior avoidance of expression and disclosure of feelings may have been used as a self-protective communicative strategy. This emphasised the poignancy of Max’s discourse in session ten; “I’ve felt guilty sometimes you know … I shouldn’t really say this but I will… the things is with me like I’m always placing all [my] trust in the relationship you know what I mean” (session ten p. 37 line 31). The frequent use of the singular first-person pronoun “I” accomplished heightened reflection, ownership of feeling and mental autonomy, although cautiously with the employment of the disclaimer “I shouldn’t really say this” and two appeals for listener endorsement, “you know.” Nevertheless, this contrasted the briskness of turns within initial sessions.

In summary, Max developed strategies for managing disagreement and showed an increased tendency to re-focus following concentration failure. Growth within the Theory of Change links increased attention and openness, which is in keeping with Max’s willingness to disclose feelings. These discursive strategies showed Max’s heightened engagement over the duration of sessions.

## Discussion

Archetypes of interactional achievement across Shared Reading sessions were presented through psychological discourse analysis. Certain rhetorical strategies were identified and their effects were characteristic of, but, importantly, not exclusive to, certain individuals and build upon both the discursive and non-discursive existing literature.

Broadening of capacity to consider different interpretations across sessions was illustrated through Clive. The function of first-person personal pronouns transitioned from predominantly establishing consensus through speaking on behalf of the group to promoting collegiality. This is reflective of research finding that the flexible use of “we” creates a power dynamic in the representation of subgroups (Kvarnström and Cedersund, 2006) and can function to construct collectivity (Sneijder et al., 2018). There was evidence for discourse shifting from speaker to listener-focussed; initial discourse, characterised by appeals to the listener and polysyndeton, contrasted with later use of non-directive substantive questions. This is in keeping with Lapadat’s (2007) finding that posing questions can promote coherence and be forward structuring. However, the current study’s findings suggest this effect may not be achieved if the language of substantive questions is directive and knowledge-testing as opposed to knowledge-seeking. Over the duration of the intervention, Clive’s discourse also showed a heightened propensity to utilise hedging, ascribing less certainty to claims.

An increase in assertiveness across sessions was identified through Patrick’s discourse. This was partly achieved by the movement from second-person plural pronouns to singular first-person pronouns to convey experience. This supports “I” functioning to narrate a personal story (Lenard, 2016) and contrasts the use of “we,” found to introduce ambiguity with respect to an agency (Jalilifar and Alavi, 2011). Increased inclination to voice disagreement contributed to greater assertiveness within the discourse of later sessions. The relationship between managing disagreement and assertiveness may be bidirectional or mutually reinforcing given that Lapadat (2007) reported that feelings of empowerment resulted from the expression of beliefs within a safe communication space. Additionally, reduced negatively inflected humour over time resulted in more positive sentiment, which can create a more positive atmosphere (Sneijder et al., 2018).

Discursive devices employed by John represented changes in self-presentation/self-disclosure. Discourse was initially characterised by repetition and alignment. This reinforced other speakers’ discourse, avoided voicing an opinion that departed from the perceived norm, and reduced accountability for discourse. Pagliai (2012) suggested that a function of non-alignment in footing was to conceal disagreement with other speakers. However, the strategy, in this case, may have also served to conceal agreement with a statement creating discordance between actual and desired self-image. Congruently, disclaimers functioned to protect the speaker from presenting a negative self-image. The movement from predominant repetition to evasive strategies and use of disclaimers achieved less explicit avoidance of expression of opinion.

Max’s non-verbal and verbal communication generally expressed a more positive attitude toward the sessions and engagement over time. Increased preferred responses within adjacency pairs and enhanced social negotiation allowing disagreement achieved inter-subjectivity. Whilst Berglund (2009) suggested that disrupted turn agency does not always lead to incoherent interaction, within this context, dis-preferred responses tended to diminish relation, leading to tangential talk that disrupted focus. Development of interactional accomplishment within the group was demonstrated through increased emotional disclosure overtime. Whilst sometimes prompted by identification with the reading material, increases in this communicative strategy were also likely to occur due to other group member’s discourse eliciting reciprocating responses and the development of familiarity and trust within the group over time.

In keeping with the Theory of Change (Davis et al., 2016), participants demonstrated a shift from “stuckness” through expanding discursive strategies employed to accomplish social action. For example, there was an increased tendency for the use of listener-focussed language and preferred responses and reduced negatively inflected humour and avoidance strategies. These cases highlight how discourse can illustrate change and indicate readiness to accept learning and self-development. Characteristics of participant talk were determinable from the start of the intervention. It is, however, noteworthy that, whilst Shared Reading interventions within other populations have demonstrated effects following six weeks (Longden et al., 2015) changes within participant discourse for the current study were discernible from around six months. This is reflective of the gradual development of sessions, the poor concentration and impulsivity of some participants, and willingness to engage with and then discuss the material.

Findings should be interpreted with consideration to study limitations. First, it is not possible to strongly assert that the changes illustrated in the discourse analysis are due to Shared Reading because participants had been receiving care and treatment, including medication and psychological therapy, at Ashworth Hospital for considerable lengths of time. One participant explicitly communicated that they had been at Ashworth Hospital for five years and two participants were preparing to transfer service toward the end of the intervention. Impracticalities and ethical issues render the elimination of many confounding factors difficult. Therefore, future research should employ a matched subjects design to assess the effects of a comparator intervention on discourse.

Additionally, it may be useful to investigate whether a similar expansion of linguistic devices persists in a female sample given that, controversially, linguistic elements may be stylistically stigmatised, associated with gender, age, and social status (Müller, 2005). A larger, more diverse sample would be required to determine the transferability of the current study’s findings and learnings. However, recruitment and implementation of a study such as this pose considerable logistical challenges. Furthermore, generalisability has been deemed a controversial topic in qualitative research; with the intentions to investigate a particular phenomenon in-depth, greater importance is often placed on the understanding of circumstances as opposed to producing representative data.

In an attempt to account for confounds, measures of therapeutic alliance, facilitator experience, participant motivation (both degree of motivation and specific reason), personality trait scores, symptomology, and changes in medication that may affect concentration and/or vocal production should be recorded. Whether changes in discourse over time are mirrored in participant’s social interactions outside the sessions could also be usefully investigated. This may also elucidate dynamics between participants outside the sessions.

Participants who dropped out of the intervention tended to be younger and at an earlier stage of illness than regular participants. Reasons for withdrawal were not pursued for ethical reasons, but voluntary feedback indicated that this was likely related to anxiety about being in a group, being recorded, concentration or interest. This may indicate that, within forensic settings, Shared Reading may be best suited to operate in tandem with or after some experience of therapy. Whilst it may be worth investigating the implementation of a Shared Reading group on a high dependency ward, it should be recognised that this environment is less conducive to undisturbed, confidential discussion and raises serious issues for audio and video recording in terms of research activity.

Overall, participant discourse strategies over the duration of the intervention showed increasingly sophisticated social function through broadening of capacity to consider, assertiveness, avoidance strategies, and engagement. The current study’s findings have practical implications for facilitators of therapeutic activity and group members. These results could be used to assess and develop criteria for interactional progress through signalling key areas for anticipated change in discourse. For example, lists of verbal expressions related to humility, assertiveness, engagement, and evasion could be developed and values assigned to assess linguistic change across therapeutic sessions, through either computerised or manual scoring.

Supporting participants to establish methods for conveying opinion or managing disagreement through the use of colloquial, as opposed to medical or therapeutic discourse, may develop “trusted” pathways of interaction that can be readily employed within day-to-day interaction in the outside world or other institutional settings. Heightened linguistic richness may also lead to greater receptiveness and responsiveness to therapy for those experiencing mental health issues, equipping individuals with a greater linguistic repertoire to explore their own narratives, in doing so promoting social activity and skills. Such changes within interaction and social behaviour can lead to cumulative changes in well-being.

## Data availability statement

The data for this study is not readily available due to their sensitive nature and for privacy reasons. Figures can be accessed for session extracts. Requests to access the datasets should be directed to MW, watkinm3@lsbu.ac.uk.

## Ethics statement

The studies involving human participants were reviewed and approved by North West—Liverpool East Research Ethics Committee (Reference 17/NW/0114). The patients/participants provided their written informed consent to participate in this study.

## Author contributions

KN and MW facilitated the Shared Reading sessions. MW conducted the analysis, informed by consultation and debrief sessions with KN and input from the wider research team. MW produced the manuscript. RC and KN reviewed the manuscript. All authors contributed to the article and approved the submitted version.
